# Early neurocognitive rehabilitation in critically ill patients during ICU stay: a safety study

**DOI:** 10.1186/2197-425X-3-S1-A994

**Published:** 2015-10-01

**Authors:** S Fernandez-Gonzalo, M Turon, G Gomà, M Martínez-Pérez, C De Haro, J Montanyà, M Jodar, J López Aguilar, L Blanch

**Affiliations:** Institut Universitari, Universitat Autònoma de Barcelona, Research Department, Fundació Parc Taulí, Sabadell, Spain; Hospital Universitari, Universitat Autònoma de Barcelona, Sabadell, Barcelona, Spain, Critical Care Department, Parc Taulí Sabadell, Sabadell, Spain; Centro de Investigación Biomédica en Red de Enfermedades Respiratorias (CIBERES), Instituto de Salud Carlos III., Madrid, Spain; Hospital Universitari, Universitat Autònoma de Barcelona, Neurology Department, Parc Taulí Sabadell, Sabadell, Spain

## Introduction

Critical illness result in significant long-term neurocognitive impairments that may persist for years after hospital discharge [1, 2]. These sequelae impact negatively in relatives' and patients' quality of life [3]. However, neurocognitive rehabilitation rarely occurs after critical illness. The Early Neurocognitive Rehabilitation in Intensive Care-ENRIC protocol (ClinicalTrial: NCT02078206)- has been develop to apply neurocognitive stimulation in patients during ICU stay. This intervention includes neurocognitive stimulation exercises that can be performed from the patient's bed through Kinect technology and it is targeted to ameliorate neurocognitive outcomes at short-and long-term.

## Objective

The aim of this study was to explore if the neurocognitive stimulation during ICU stay can produce deleterious effects over physiological status.

## METHOD

18 ICU participants received a 20 minutes Early Neurocognitive Rehabilitation session. Heart rate (HR), O_2_ saturation (SpO_2_) and respiratory rate (RR) were collected using BetterCare^®^ system 20 minutes before, during and after the neurocognitive stimulation session. Safe ranges were calculated by age and sex and age in HR and RR respectively. Safe lower SpO_2_ limit was estimated at 90%. As safety criteria safe ranges and a change of 20% from baseline in any physiological parameter were considered.

## Results

100% of the sample presented HR values within normal limits at baseline (Mean= 92.3; min-max: 62.7-120.8), during (93.2; 75.6-120.7) and after session (93.4; 83.4-118.2). SpO_2_ values at baseline (95.7; 91.5-99.9), during (95.4; 91.8-100) and after session (95.7; range: 92.7-100) were within normal limits in all participants. 9 participants exceeded safe RR limits at baseline (27.6; 21.4-34), maintaining this status during (27.2; 22.1-34.3) and after session (27.9; 22.4-33.5). No changes greater than 20% were observed in any case. 2 new patients exceeded safe RR limits after session (26.5; 20.8-32.2), although only 1 participant showed a change greater than 20% (21.9%). No significant pre vs. post results were observed in any physiological variable (HR, p = 0.85; SpO, p = 0.98; RR, p = 0.23).

## Conclusions

Neurocognitive stimulation in patients during ICU stay did not produce clinical relevant changes in the patients' physiological status. Thus, the early neurocognitive stimulation may be considered a safety intervention for critically ill patients.

## Grant Acknowledgment

Fundació La Marató TV3
